# MOF-Based Sorbents Used for the Removal of Hg^2+^ from Aqueous Solutions via a Sorption-Assisted Microfiltration

**DOI:** 10.3390/membranes12121280

**Published:** 2022-12-17

**Authors:** Agnieszka Miśkiewicz, Wojciech Starosta, Rafał Walczak, Grażyna Zakrzewska-Kołtuniewicz

**Affiliations:** Institute of Nuclear Chemistry and Technology, Dorodna 16, 03-195 Warsaw, Poland

**Keywords:** metal-organic-framework compounds (MOFs), sorption-assisted microfiltration (SAMF), mercury removal, water purification

## Abstract

Mercury is considered to be one of the most important chemicals of public health concern. Therefore, it is necessary to develop an effective method of removing mercury ions from aqueous solutions to protect people from exposure to this element. This paper presents research on the application of a sorption-assisted microfiltration (SAMF) hybrid process for the removal of Hg^2+^ from aqueous solutions. As adsorbents used in the process, the metal-organic-framework-UiO-66-type materials have been considered. The methods of synthesis of two types of metal-organic-framework (MOF) sorbents were developed: UiO-66_MAA modified with mercaptoacetic acid (MAA) and a composite of UiO-66 with cellulose. The results of the experiments performed proved that the separation of Hg^2+^ from water solutions conducted in such a system was effective; however, a relatively long initial contact time of reagents before filtration was required. The experimental results can be used to optimize the parameters of the SAMF process in order to obtain an effective method of Hg^2+^ removal from aqueous solutions.

## 1. Introduction

The World Health Organization (WHO) has listed mercury (Hg) as one of the ten pollutants of particular concern to public health. Mercury can be released from several sources, such as electronics, paints, pharmaceuticals, paper and pulp, chlor-alkali, oil refinement, plastics, rubber processing, and manufacturing industries. Other significant (>10%) sources of Hg emission include coal and other fossil fuel combustion (25%), and the production of non-ferrous metals. Moreover, mercury compounds can persist and accumulate in the environment, causing severe toxicity to humans and animals, especially methylmercury. The fate of inorganic mercury ions in nature is that they turn into methyl mercury due to the aerobic action of microorganisms.

There are various methods of removing heavy metals, such as mercury, from an aqueous environment, which include reverse osmosis, chemical precipitation, ion exchange, coagulation, and adsorption [1,2,3,4,5]. There are many novel processes considered for heavy metal ions removal from aqueous solutions [6,7]. However, adsorption appears to be the best-suited process for this purpose due to its simplicity and cost efficiency. The development of a sorbent for the removal of mercury contaminants from aqueous solutions is associated with the selection of the type of sorbent and the method of installing a functional group showing an affinity for mercury within it. Such properties are demonstrated by MOF materials made of high valence metals with, in particular, the zirconium terephthalate structure, known in the literature as UiO-66 [8].

Porous materials of the MOF type have been of great interest for about thirty years. The reason for such a large and still-growing interest are the unique properties of these materials, the multitude of possible structures that can be obtained, the relatively simple method of their synthesis and emerging applications [9,10,11,12]. MOF structures are built of small metallic or oxygen-metallic groups connected with each other by coordination bonds by means of organic ligands with at least two functional groups, mainly carboxylate groups. The possibility of independently shaping the form of the organic and inorganic parts makes it possible to obtain theoretically unlimited numbers of structures differing in the porosity parameters: pore geometry, the value of the specific surface area of the pores, and the physicochemical properties of the pore walls. The research carried out so far indicates a wide range of applications in the area of gas sorption and storage, separation of gas mixtures, encapsulation of medical preparations, and the removal of heavy metals and radioactive isotopes from aqueous solutions, in the area of electrode materials for modern batteries, etc. [13,14,15,16,17,18,19]. The number of MOFs deposited in the CSD database is currently 111570 (Cambridge Structural Database v. 5.42 of November 2022). However, only a few known MOFs have found practical application and reached the level of a commercial product [20]. The critical factors limiting implementation can be manifold, such as the lack of commercially available linker or a high linker price, the lack of cost effective synthesis procedures, and insufficient studies to demonstrate the suitability of the material. In most cases, implementation works were limited to a laboratory scale using small tenths-of-a-gram samples. Their synthesis procedures have not been optimised for large-scale production. This applies in particular to the use of an environmentally toxic solvent such as dimethylformamide (DMF) that is not recommended for large-scale production.

The unique features of MOFs, such as a large specific surface area, the diversity of possible structures, and the theoretical possibility of a design structure for specific applications make them suitable for industrial applications. However, extensive application research is required before a potentially beneficial solution can be put into practice. The issue of economic efficiency and technical suitability should be resolved first. 

This paper presents the results of a study on the removal of mercury from aqueous solutions using an MOF-based sorbent in a hybrid sorption–microfiltration process. Hybrid methods, in which one of the processes is membrane filtration, are widely used (e.g., in various industries, and for environmental protection) [21,22] and valued due to the number of their advantages, which include low energy consumption, high removal efficiency, and the possibility of conducting two processes (sorption and filtration) in one apparatus, which reduces the operating costs of the entire process. Membrane-based hybrid processes were proved to be promising for the removal of traces of metallic ions from aqueous solutions [23,24,25,26]. One such method, complexation–ultrafiltration using polyethylenimine (PEI) as a complexing agent, was studied for the removal of mercury ions from wastewater [27]. The results showed that for neutral solutions mercury retention values close to 100% can be obtained.

## 2. Experimental Methods

### 2.1. Chemicals

All chemicals used in this work were of analytical-grade purity. Zirconium chloride (ZrCl_4_), mercury chloride (HgCl_2_), terephthalic acid, N,N-dimethylformamide and microcrystalline cellulose were purchased from SIGMA-ALDRICH and were used as received without any further purification.

The radionuclide of mercury-197 (radioactive half-life, t_1/2_ = 64.14 h; energy of emitted radiation, Eγ = 69 keV), was obtained by neutron activation of mercury nitrate of natural composition in the MARIA nuclear research reactor in Świerk, Poland and has been used as a radiotracer in this research.

### 2.2. Analytical Methods

The microscopic morphological studies were performed using the Zeiss Ultra Plus (Zeiss, Jena, Germany) High Resolution Scanning Electron Microscope (SEM). For the elemental composition determination of micro-samples, the Quantax 400 (Bruker) Energy-Dispersive Spectrometer (EDS) for SEM was used. The X-ray diffraction (XRD) spectra were collected with the D8 Advance X-ray diffractometer equipped with Cu tube and Bragg-Brentano optics with fixed slits, Ni filter, and LynxEye SSD160-2 position sensitive detector. A home-made X-ray fluorescence spectrometer (XRF) with Rh X-ray tube for fluorescence radiation excitation, Si detector and MAESTRO software for spectra collection were utilised for the determination of molar Hg/Zr ratios on sorbent samples obtained by filtration on a track-etched membrane. The total concentrations of radionuclides in the permeate samples were determined using a gamma counter (LG-1b type, INCT, Poland). 

### 2.3. Synthesis and Characterisation of the MOF-Type Sorbents

The synthesis procedures of two types of sorbents for mercury ions– UiO-66_MAA and their composite with cellulose sorbent were developed and described below.

#### 2.3.1. Synthesis of MAA Sorbent

**Figure 1:**
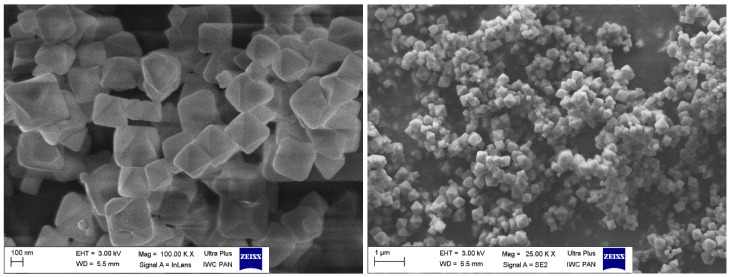
SEM images of synthesised sorbent.

The synthesis was conducted by refluxing the solution of zirconium chloride (4 mmol), terephthalic acid (4 mmol), and mercaptoacetic acid as a modulator in DMF solvent (100 mL) at 120 °C for 24 h. The process was carried out with the Carouselle-6, parallel synthesis system enabling simultaneous synthesis in six 250 mL flasks. The synthesis results in 7 g of UiO-66 type structure as confirmed by X-ray diffraction that gives near-100% efficiency. Five modulator concentrations equal to 10, 20, 50, 70, 90, and 100 equivalents of zirconium, respectively, were tested. The successful incorporation of –SH groups into UiO-66 structure was confirmed by EDS analysis in all cases. The ratio of molar S/Zr content was determined as equal to 0.21 for the case of 10 eq mercaptoacetic acid (MAA) modulator content, 0.32 for 50 eq, and 0.32 for the case of 100 eq. For further studies, the concentrations of the modulator have been fixed to 50 eq of zirconium. The SEM images presented in Figure 1 show that synthesised material consists of intergrown aggregates of small size crystallites with dimensions of around 100 nm.

**Figure 2:**
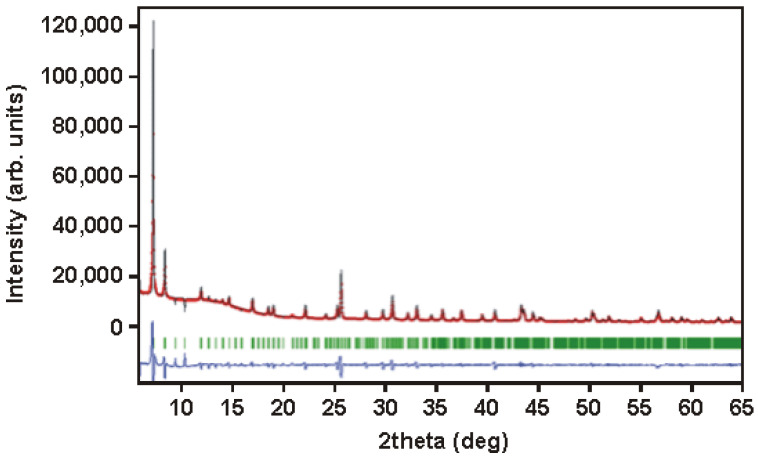
The diffraction pattern of synthesised material and the result of their refinement with tetragonal cell a=b=10.38 Å and c= 23.96 Å. The blue line represents the difference of the patterns.

The diffraction spectrum measured with D8 Advance diffractometer, shown in Figure 2 confirms the crystalline nature of synthesised material. The observed diffraction pattern fairly accurately resembles that of zirconium terephthalate MOF cubic structure known in the literature as UiO-66 [28]. However, the detailed analysis of the observed pattern shows the possibility of the tetragonal unit cell with a higher volume and parameters of unit cell a=b=10.38 Å and c= 23.96 Å. The result of the fitting of the observed spectrum using Fullprof Suite software and parameters of this tetragonal cell are also shown in Figure 2. The search for a solution is the subject of this research and seems to be possible on the basis of measurements of the powder spectrum at the synchrotron.

#### 2.3.2. Synthesis of MOFs Composite Materials with Cellulose

MOF materials are usually obtained as powders with a size of tens of nanometres. Small particle sizes may be a problem in certain applications as this may affect the rheological properties of the liquid medium or be responsible for particle leaching in the case of column filtration. These deficiencies can be avoided by the synthesis of MOFs with composite materials, e.g., with cellulose.

**Figure 3:**
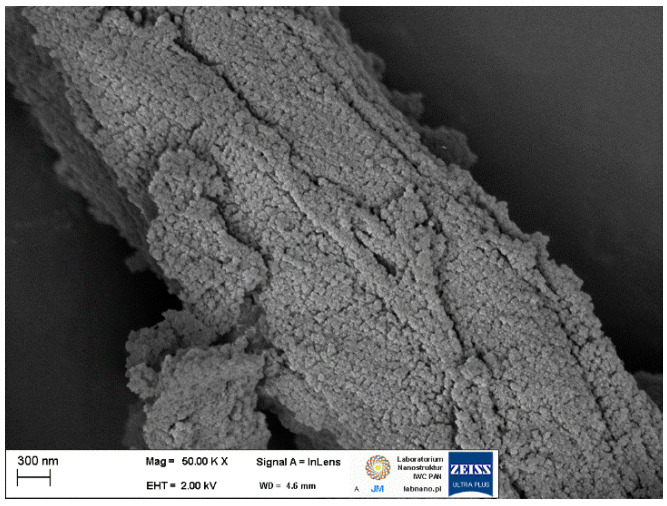
SEM image of small-sized UiO-66-type crystallites of MOF deposited on the cellulose fibre.

In the present experiments, UiO-66 and cellulose composites using microcrystalline cellulose offered by SIGMA-Aldrich were synthesised. The synthesis process consisted of two steps. In the first one, carboxylate groups were grafted onto the cellulose surface using succinic anhydride. In a typical synthesis, 0.5 g of microcrystalline cellulose was dispersed in 50 mL DMF and 1 g of succinic anhydride and 1 mL of pyridine were added to the suspension. The mixture was processed for 12 h at 80 °C. Material recovered by filtration was suspended in a solution of zirconium chloride, terephthalic acid, and mercaptoacetic acid in DMF and processed further at 120 °C for 24 h. The SEM studies presented in Figure 3 confirm the deposition of small crystallites on the surface of cellulose fibre.

XRD diffraction studies confirmed the presence of both materials in the composite. However, the diffraction peaks were broadened significantly. The size of crystallites was estimated as 17 nm on the basis of the Debye–Schere formula:

(1) $$d=\frac{k\cdot \lambda }{\beta \cdot cos\theta }$$

where *d* is the size of the particle, *k* is Scherer’s constant (*k* = 0.94), *λ* is the X-ray wavelength (1.54178Å), and *β* is the full width at half maximum (FWHM) of the diffraction peak.

### 2.4. Sorption of Mercury Ions in Batch System

The sorption studies were carried out in a batch mode using 10 mg of sorbent suspended in 10 mL of mercury chloride solution with varying concentrations of mercury in the range of 0.5 to 10 mmol. The mixture was shaken for two hours. After that, it was filtered using a 0.2 µm track-etched membrane filter. The sorbent deposited on the membrane was analyzed for mercury and zirconium content using an X-ray fluorescence spectrometer with X-ray tube excitation. Analysis of the collected spectra after calibration allowed us to determine the mercury-to-zirconium molar ratio on the filter.

### 2.5. A Sorption-Assisted Microfiltration Process

In a sorption-assisted process, a tubular membrane module with the Couette-Tylor Flow (CTF) was used. CTF is a combination of the axial Poiseuille flow and the rotating Couette flow with axisymmetric Taylor vortices. Such a combination results in limited axial dispersion coefficients in relation to dispersion coefficients in other directions, an independence of mixing intensity on the residence time of the medium in the apparatus, and good transport parameters [29]. This flow arrangement also reduces membrane fouling which is a problem in membrane installations.

**Figure 4:**
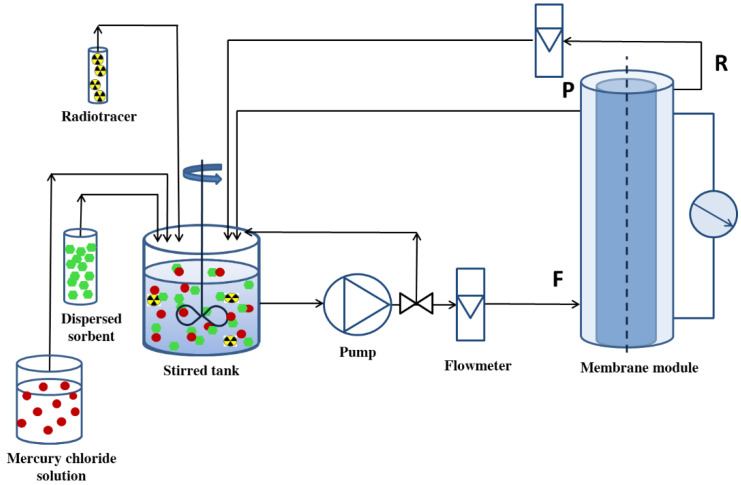
Experimental set-up used for sorption-assisted microfiltration experiments.

The experimental set-up is schematically presented in Figure 4. The tubular membrane used in the installation was made by sintering of metals, such as: 16–18% Cr, 10–14% Ni, 2–3% Mo, and 65–72% Fe. The membrane pore size was 0.1 μm, and an effective membrane area of 0.04 m^2^ was used. Inside the membrane, in which the inner and outer diameter was equal to 30 mm and 34 mm, respectively, a roller-shaped rotor with the diameter of 20 mm was placed. This configuration of the membrane module allows the CTF flow formation during the filtration.

Feed solutions for filtration experiments were prepared as follows: to the 2L of stock solution of 0.01 M Hg^2+^ an appropriate amount of MOF sorbent was added followed by the addition of a small amount of radiotracer solution (^197^HgCl_2_). In this work, the radiotracer method was used in order to assess the efficiency of Hg^2+^ sorption on MOF and the efficiency of retention of these ions on the membrane. Radioisotope tracers can be used at very low concentrations (as radiation detectors are very sensitive), without disturbing the concentration of the ions of interest.

Prior to the filtration experiment, the pH was adjusted with NaOH and the feed solution was stirred for 60 or 120 min (experimentally determined to be a sufficient time for Hg^2+^ ions adsorption on MOF particles). Then the mixture of reagents was used as a feeding solution for the membrane installation. Process parameters were as follows: transmembrane pressure (*p*): 0.5 bar; feed flow rate (*Q_F_*): 1.0 L/min, and rotation frequency of the inner shaft (*Ω*): 1000 rpm. The process was carried out in a closed system, meaning that the permeate (P) and retentate (R) streams were returned to the feed tank after exiting the membrane module. During filtration, permeate samples were collected periodically and analysed in relation to the Hg^2+^ content. The Hg^2+^ contents were determined by measuring the radioactivity of the ^197^Hg radiotracer using the gamma counter LG-1b. The retention coefficient (*R)* was then calculated according to Formula (2):

(2) $$R=\left(1-\frac{A_{P}}{A_{F}}\right)\cdot 100\%$$

where *A_P_* and *A_F_* represent the activity of radiotracer in the permeate and feed, respectively, counts per second, cps.

Sorption capacity (*q*) of MOF-type sorbents was calculated as follows (3):

(3) $$q=\frac{\left(\frac{R}{100}\cdot m_{Hg_{0}}\right)}{m_{sorb.}}$$

where *R* is the retention coefficient of Hg ions, %, *m*_*Hg*0_ is the initial mass of Hg ions in 2 L of stock solution, mg and *m_sorb_*_._ is a mass of sorbent used for the preparation of 2 L of stock solution, g.

## 3. Results and Discussion

### 3.1. MOF as a Sorbent for Mercury Ions

**Figure 5:**
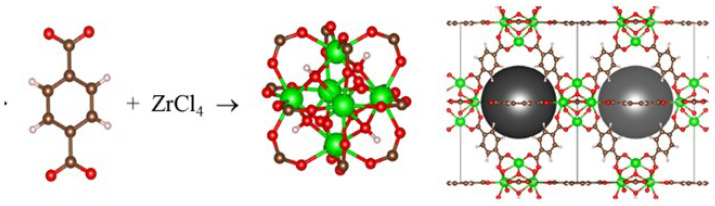
The image of the structural building units and crystal structure of UiO-66 MOF showing the octahedral pores (green depicts Zr, red depicts oxygen, and black depicts carbon).

The development of a sorbent for the removal of mercury contaminants from aqueous solutions is associated with the selection of the type of sorbent and the method of installing a functional group showing an affinity for mercury within it. Due to the nature of work in an aquatic environment, it is necessary that the sorbent is resistant to water. Such properties are demonstrated by MOF materials made of metals with high valence, in particular the zirconium terephthalate structure, known in the literature as UiO-66 [8]. The basic structural building unit of this material is the octahedral cluster of six zirconium cations, which was created using the VESTA program [30] and as shown in Figure 5. The octahedron walls of the cluster are capped alternately with four oxygen anions and four hydroxyl groups. The role of the linker in the original structure is played by the anion of the commonly available terephthalic acid. The chemical structure of UiO-66 is described by the formula Zr_6_O_4_(OH)_4_(BDC)_6_, where BDC stands for double-ionised terephthalic acid anion.

The structure of UiO-66 is characterised by a significant specific surface area of pores, around 1600 m^2^/g, and a pore volume of 0.70 cm^3^/g. An important feature of this structure is its ability to accommodate the structural microscopic defects, such as missing ligands or missing metal clusters. According to the literature data, it is possible to replace the original terephthalate linker (up to two linkers from six per structural unit) with a pair of monocarboxylic anions (formic acid, acetic acid) or a combination of these ligands, a hydroxyl group and water molecule, provided that the charge neutrality of the system is conserved. This property enables the chemically inert UiO-66 structure to obtain new functionalities, in particular, the affinity to sorbate. For that purpose, a so-called modulated synthesis method is applied, consisting of an addition to the synthesis mixture of a monocarboxylic ligand possessing a non-coordinating functional group having an affinity for sorbate [31,32,33]. According to the HSAB theory for the coordination of mercury which is a soft acid, a suitable soft base is required. The sulfhydryl group –SH fulfils this requirement very well, as documented in the literature [34,35,36]. Mercaptoacetic acid (MAA) and mercaptosuccinic acid (MSA) are the simplest commercially available ligands possessing the sulfhydryl group necessary for the functionalisation of UiO-66 used in these studies.

### 3.2. Sorption of Mercury Ions on MOF Material in Static Mode

**Figure 6:**
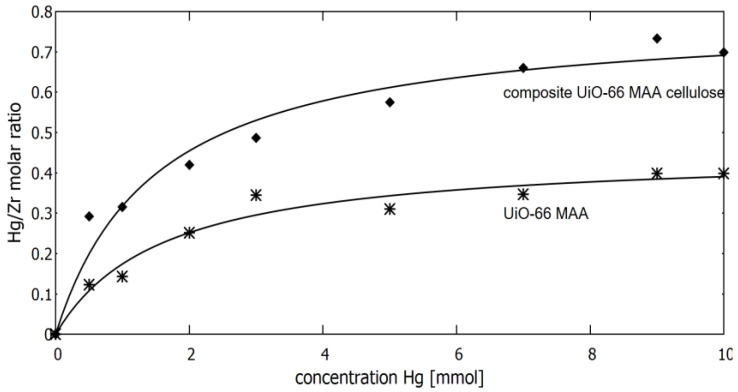
Sorption isotherms and their approximation with the Langmuir model for UiO-66_MAA sorbent and their composite with cellulose.

For the static mode determination of sorption properties, 10 mg of sorbent was added to 10 mL of mercury chloride water solution with different concentrations of mercury in the range of 0.5–10 mM. The resulting suspension was equilibrated for two hours under continuous stirring. After that, the sorbent was separated by filtration on a track-etched membrane with a 0.2 µm pore diameter and analysed for the molar ratio of mercury to zirconium content using the X-ray fluorescence method. The results of the experiments showing dependence of the molar ratio of mercury-to-zirconium on the concentration of mercury for both sorbents used in experiments (zirconium UiO-66_MAA and their composite with cellulose UiO-66_MAA_cellulose) are presented in Figure 6. The experimental data were approximated with a Langmuir model using the nonlinear regression method. For the UiO-66 sorbent modified with MAA, the calculated sorbent capacity after two hour’s of contact time, expressed as the molar ratio of mercury accumulated on sorbent to zirconium, reached a value of 0.46 ± 0.03 (or 332 mg/g calculated after taking into account the molar mass of mercury and UiO-66 sorbent). In the case of their composite with cellulose, a higher value for the molar Hg/Zr ratio equal 0.79 ± 0.05 was obtained. The reason for this difference is probably due to the different morphologies of the sorbents e.g., the significantly smaller size of UiO-66 crystallites in the case of the cellulose composite material.

**Figure 7:**
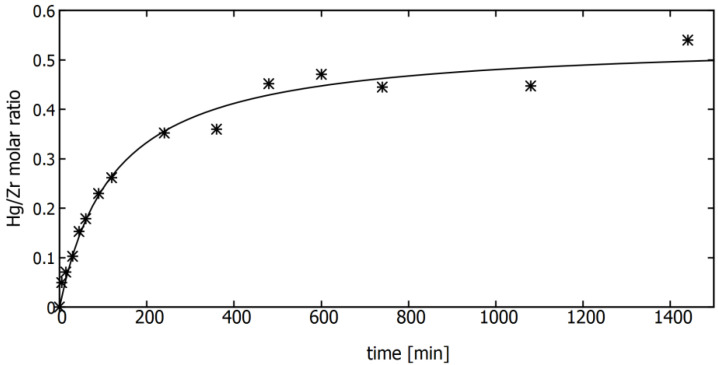
The kinetics of sorption on the UiO-66-type sorbent functionalised with mercaptoacetic acid and its approximation with the second-order kinetic model.

For the kinetic properties of the UiO-66_MAA sorbent studies, 10 mg of sorbent and 10 mL of water solution of mercury chloride with a concentration of mercury of 1 g/L were equilibrated for a predetermined time in the range of 0.5 min to 24 h. The results expressed as a molar ratio of mercury accumulated on sorbent to zirconium, determined by the XRF method on the samples recovered by filtration are shown in Figure 7. The experimental data were approximated using the second order kinetic model. This model has been selected at the first step due to the high value of R^2^ coefficient (equal to 0.9856) obtained in approximation of the experimental points using a pseudo second order model linear form. The value of 0.54 ± 0.01 for the molecular Hg/Zr ratio was obtained for the sorbent capacity and the value of 125.12 min for the half-life time (the time required to consume half of the starting concentrations of the adsorbate).

**Figure 8:**
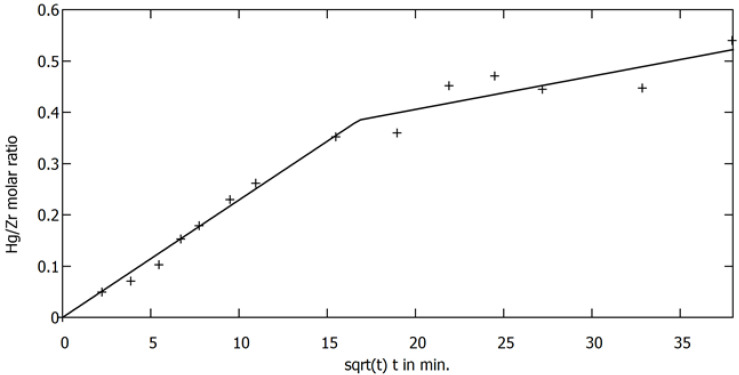
The dependence on the amount of mercury accumulated on the sorbent as a function of the square root of time.

However, the systematic deviation of experimental points from linear curve in the linearised model were observed for the small time values. Looking for an alternative model, we turned our attention to an intraparticle diffusion model [37]. This model seems to be better suited to describe elementary processes occurring during sorption on porous materials. In the present case, mercury diffuses across the pore system of the sorbent to the active -SH sulfhydryl groups located on the pore walls, where it is chemically bound. The requirement of the model experimental data were presented as a function of the square root of time in Figure 8. It can be seen that experimental points can be approximated with two piecewise linear functions. The first one passes through the origin of the graph (0,0) and R^2^ coefficient for the approximation of this part of the graph with linear function is equal to 0.9907. It gives strong support for the validity of intraparticle diffusion model. However, two different mechanisms of the diffusion can be discerned. A detailed description of the sorption model will be the subject of a separate study.

### 3.3. Sorption-Assisted Microfiltration for Mercury Ions Removal

The MOF-assisted microfiltration process for mercury removal was performed using two types of sorbent synthesised according to the procedure described in Section 2.3.1 and Section 2.3.2. The influence of the process parameters on the mercury ion removal efficiency has been evaluated and the two sorbents (UiO-66_MMA sorbent and composite sorbent) were compared in terms of Hg^2+^ removal efficiency.

**Figure 9:**
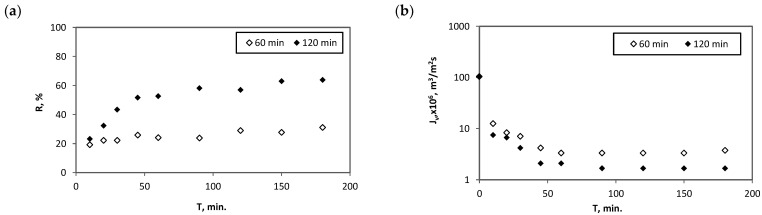
Changes in retention coefficient (**a**) and permeate flux (**b**) during sorption-assisted MF process in the case of experiments where the effect of increasing the initial contact time of the reagents was analysed; UiO-66_MAA sorbent, C_sorb._ = 1 g/L.

The influence of the time of mixing the reagents before filtration, i.e., initial contact time of the reagents (T_cont._) on the retention coefficient of Hg^2+^ is illustrated in Figure 9a. As can be observed, the increase in the initial contact time allows for an increase in the Hg^2+^ removal efficiency—the coefficient R achieved at the end of the process for a longer mixing time reached a value of 64% while for a shorter time R amounted to 31%. At the same time when permeate samples were collected and analysed in terms of Hg content, a permeate flux (J_v_) was measured. The results of these measurements for experiments described above are presented in Figure 9b. As expected, an initial sharp decrease in J_v_ was observed followed by a gradual stabilisation of the flux at a constant level of about 1.6–3.3 × 10^−6^ m^3^/m^2^ × s. No significant change in the J_v_ is observed with the initial contact time of the reagents being extended.

Values of the sorption capacity (q) of UiO-66_MAA sorbent, calculated using formula (3) for 60 and 120 min of initial contact time were: 622 mg/g and 1282 mg/g, respectively. Summarising the obtained results, it should be emphasised that, extending the contact time of the reactants before filtration has a positive effect on the sorption capacity of UiO-66_MAA sorbent, and thus on the increase in the Hg^2+^ retention coefficient.

The influence of the pH and reagents ratio on the retention coefficient (R) of Hg^2+^ ions using UiO-66_MAA sorbent was also determined. A detailed discussion of these experiences and their results has been described elsewhere [38]. The highest retention coefficients were obtained for the ratio of the reagents equal to 1/1 (R = 86%). However, due to the possibility of significant membrane fouling in the case of using high concentration of a sorbent, it was decided to conduct further research at its average concentration, 1 g/L, which gives the reagent ratio (Hg/MOF) equal to 2/1.

**Figure 10:**
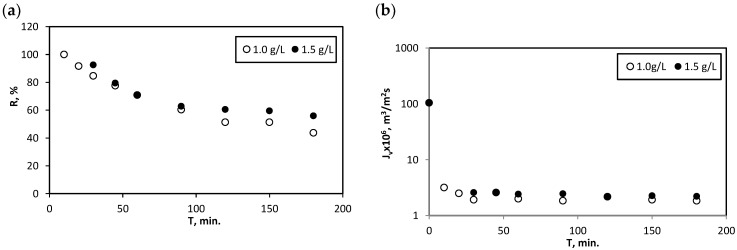
Changes in retention coefficient (**a**) and permeate flux (**b**) during sorption-assisted MF process in the case of experiments where the effect of increasing sorbent concentration was analysed; composite MOF-type sorbent; T_cont._ = 60 min.

The next stage of the research was the analysis of the impact of reagent ratio increase on the efficiency of Hg^2+^ removal in the case of the use of composite MOF-type sorbent in the SAUF process. For this purpose, two different sorbent concentrations were applied: 1.0 g/L (which gives 1/1 reagent ratio, g/g) and 1.5 g/L (which gives 3/4 reagent ratio, g/g). Unfortunately, a wider range of reagent ratio, i.e., a larger excess of sorbent concentration in relation to mercury ion concentration was not possible due to synthesis restrictions. The synthesis of larger amounts of sorbent still requires refinement. In addition, there were concerns about the intensification of membrane fouling due to increased sorbent concentration. The results of these experiments are illustrated in Figure 10a,b. As can be observed, in the examined range of changes in the reagent ratio, there is a slight increase in the mercury ion retention coefficient with an increase in the sorbent concentration (Figure 10a). This change in the sorbent concentration also did not significantly affect the permeate flux (Figure 10b), i.e., it did not increase the unfavourable phenomenon of fouling. Values of the sorption capacity (q) of composite sorbent, calculated using formula (3) for 1.0 and 1.5 g/L were: 875 mg/g and 748 mg/g, respectively.

Moreover, analysing Figure 10a, it can be noticed that R decreases during the process. This phenomenon was observed regardless of the applied process conditions. Since no simultaneous turbidity of the permeate was observed that would indicate the passage of the sorbent through the membrane, it can be assumed that during the process mercury ions may be partially leached from the composite sorbent.

**Figure 11:**
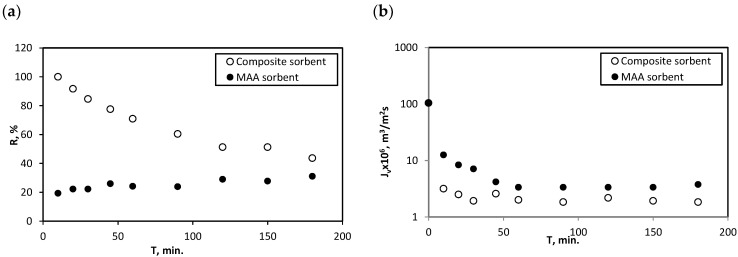
Changes in retention coefficient (**a**) and permeate flux (**b**) during sorption-assisted MF process for two types of MOF sorbents: UiO-66_MAA sorbent and composite sorbent, Csorb. = 1 g/L, T_cont._ = 60 min.

The next figure (Figure 11a,b) presented a comparison of the two synthesised sorbents in terms of retention coefficient of Hg^2+^ (Figure 11a) as well as permeate flux decline during the process of SAMF. In general, composite sorbent proved to be more effective in removing mercury ions from a water solution via SAMF process. In the case of this sorbent, higher values of R were obtained. Also, calculated capacities were higher for the composite sorbent than the UiO-66_MAA sorbent and amounted to 875 mg/g and 622 mg/g, respectively.

A decrease in R value during the process, observed for the composite sorbent, although unfavourable, did not decrease below the R value achieved for the UiO-66_MAA sorbent. However, this phenomenon of possible leaching of mercury from the sorbent requires further research.

After the completion of filtration run at the sorbent-assisted microfiltration installation, the UiO-66_MAA sorbent was recovered by filtration on the track-etched membrane. The deposit was analysed for morphology changes by SEM microscopy and for mercury and zirconium content using X-ray fluorescence spectrometry.

**Figure 12:**
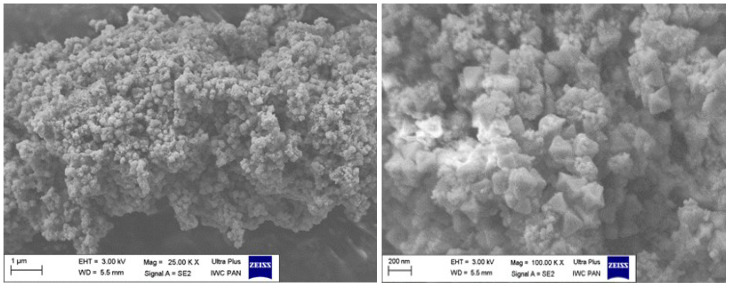
SEM images of the UiO-66_MAA sorbent after filtration at the sorbent-assisted microfiltration installation.

SEM images presented in Figure 12 showed that crystalline morphology of the sorbent had been preserved.

**Figure 13:**
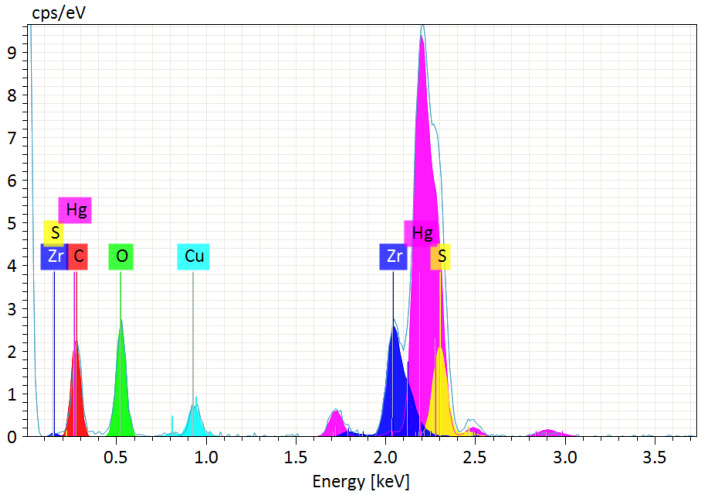
The EDS spectrum of the sorbent recovered after filtration with the sorption-assisted microfiltration installation. (The Cu lines result from the evaporated copper layer required for the microscopic analysis).

The EDS spectrum of the sorbent after filtration presented in Figure 13 confirmed the successful incorporation of sulphur into the zirconium sorbent at the synthesis step and accumulation of mercury during sorption. The percentage atomic content of the main elements in the sample was: Zr—6.29 ± 0.47%, S—7.50 ± 0.24%, Hg—13.69 ± 1.96%, C—42.30 ± 1.58%, O—30.23 ± 1.45%.

X-ray fluorescence spectrometry analysis of the samples recovered from the microfiltration device measured after a few days showed that the molar ratio of mercury to zirconium was about two. This value is 2–3 times higher than that obtained in laboratory tests based on a small amount of the sorbent equal to 10 mg and a contact time of two hours. About half of the amount of mercury accumulated on the sorbent can be removed by soaking in 4N hydrochloric acid solution. The rest of the mercury remains bound after 24 h of acid exposure.

## 4. Discussion

The conducted studies have shown that the removal of mercury in the hybrid MF process assisted with sorption using MOF sorbents is feasible and may bring technological benefits. The use of a system combining two processes in one installation is beneficial primarily in terms of reducing the capital and operating costs of the entire process. Another benefit of the described case is the choice of a membrane module with helical flow. Thanks to this solution, the hydrodynamic conditions in the membrane module contribute to an increase in mass transfer coefficients between the liquid and the membrane resulting in enhancement of the filtration efficiency as well [29].

However, several factors need to be carefully considered and taken into account when setting up SAMF process using MOF-type sorbents. As the one of process involved in the hybrid method is sorption, suitable conditions for the sorption of Hg^2+^ on the MOF-type sorbent should be ensured, i.e., the appropriate pH, contact time of the reactants as well as the Hg^2+^/sorbent ratio. As was previously revealed [38], the process of Hg^2+^ ion sorption on MOF is significantly influenced by the pH and the reagent concentration ratio. The results of the study of kinetics of Hg^2+^ sorption on the UiO-66 type sorbent, shown in Figure 7, indicated that the sorption equilibrium is established after about 20 h. Bearing in mind the abovementioned advantages of the helical flow membrane module, in particular the increase in the mass transfer coefficient, an attempt was made to shorten the contact time of the reactants to 2 h and even to 1 h (Figure 9). While increasing the initial contact time allows for an increase in the Hg^2+^ removal efficiency, the process needs further optimisation before implementation in the industry.

The positive effect of the increase in the sorbent dosage increasing the retention coefficient of Hg^2+^ also was observed. However, increasing the sorbent dosage has its limitations, one of which is related to the possibility of fouling in the case of high sorbent concentrations. In this research, the reagent ratio was 1/1 or 3/4, and under such conditions a slight effect of the sorbent dose on R was observed (Figure 10a). Moreover, such conditions did not significantly affect the intensity of the fouling phenomenon (Figure 10b).

When comparing two types of sorbent, for which a synthesis process was developed in the study, it can be noticed that the composite sorbent proved to be more effective in removing mercury ions from a water solution via the SAMF process (Figure 11).

## 5. Conclusions

Summarising the research carried out, it can be concluded that the application of the SAMF process to remove mercury ions is feasible and promising. After taking into account several important factors, such as appropriate sorption conditions, selection of a membrane with an appropriate pore size and obtaining a mechanically stable sorbent, the process can be successfully used for the removal of hazardous elements from aqueous solutions with high efficiency.

To the best of our knowledge, MOF-type materials have been used for the first time to purify water from toxic metals such as mercury on a scale that goes beyond the laboratory by using a sorption-assisted microfiltration system. The possibility of adapting the relatively inert sorbent (UiO-66), for mercury sorption through the modulated synthesis method with the use of a commonly available reagent (mercaptoacetic acid) was also demonstrated.

## Data Availability

Not applicable.
